# Supplementary material for: Prior immunity to Ureaplasma urealyticum protects against respiratory infection in immunosuppressed mice
Source: Microbiol Spectr. 2024 Nov 19;13(1):e01763-24. doi: 10.1128/spectrum.01763-24 (PMC11705917; doi:10.1128/spectrum.01763-24)
Supplement: Figure S1 — Blood ammonia concentrations in Ureaplasma urealyticum- and Ureaplasma parvum-infected control and immunized mice. [file spectrum.01763-24-s0001.pdf]

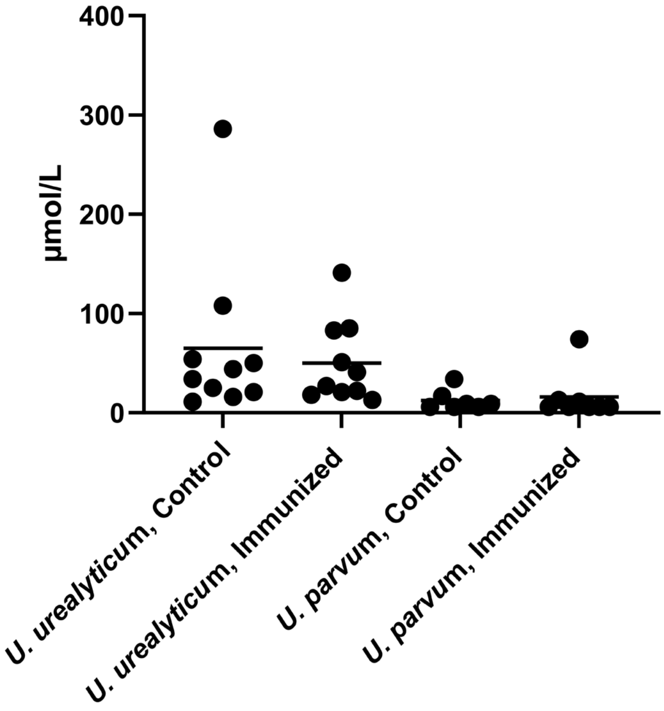

Supplementary Figure. Blood ammonia concentrations in *Ureaplasma urealyticum-* and *Ureaplasma parvum*-infected control and immunized mice, using cardiac puncture blood prior to murine euthanasia. There were no significant differences (Mann-Whitney) in blood ammonia levels between control and immunized mice with either infection protocol. GraphPad Prism 10 was used to generate statistics and to plot the graph.
